# Optimization of Microalga *Chlorella vulgaris* Magnetic Harvesting

**DOI:** 10.3390/nano11061614

**Published:** 2021-06-20

**Authors:** Maria G. Savvidou, Maria Myrto Dardavila, Ioulia Georgiopoulou, Vasiliki Louli, Haralambos Stamatis, Dimitris Kekos, Epaminondas Voutsas

**Affiliations:** 1 Biotechnology Laboratory, School of Chemical Engineering, National Technical University of Athens, 9 Iroon Polytechniou Str, Zografou Campus, 15780 Athens, Greece; msavvid@central.ntua.gr (M.G.S.); kekos@chemeng.ntua.gr (D.K.); 2 Laboratory of Thermodynamics and Transport Phenomena, School of Chemical Engineering, National Technical University of Athens, 9 Iroon Polytechniou Str, Zografou Campus, 15780 Athens, Greece; ioulia.p.geo@gmail.com (I.G.); svlouli@chemeng.ntua.gr (V.L.); evoutsas@chemeng.ntua.gr (E.V.); 3 Laboratory of Biotechnology, Department of Biological Applications and Technologies, University of Ioannina, 45110 Ioannina, Greece; hstamati@uoi.gr

**Keywords:** *Chlorella vulgaris*, microwave-synthesized magnetite particles, response surface methodology, harvesting process optimization

## Abstract

Harvesting of microalgae is a crucial step in microalgae-based mass production of different high value-added products. In the present work, magnetic harvesting of *Chlorella vulgaris* was investigated using microwave-synthesized naked magnetite (Fe_3_O_4_) particles with an average crystallite diameter of 20 nm. Optimization of the most important parameters of the magnetic harvesting process, namely pH, mass ratio (m_r_) of magnetite particles to biomass (*g/g*), and agitation speed (rpm) of the *C. vulgaris* biomass–Fe_3_O_4_ particles mixture, was performed using the response surface methodology (RSM) statistical tool. Harvesting efficiencies higher than 99% were obtained for pH 3.0 and mixing speed greater or equal to 350 rpm. Recovery of magnetic particles via detachment was shown to be feasible and the recovery particles could be reused at least five times with high harvesting efficiency. Consequently, the described harvesting approach of *C. vulgaris* cells leads to an efficient, simple, and quick process, that does not impair the quality of the harvested biomass.

## 1. Introduction

Microalgae have been extensively investigated over the past decades as a source for biofuel production due to their high lipid and carbohydrate yields, as well as being a natural source of high value-added bioactive compounds such as polyphenols, carotenoids, fatty acids, and antibiotics [1,2,3]. Bioactive chemicals derived from natural sources present higher biological activity and acceptance by consumers compared to the synthetic alternatives [4].

Nowadays, microalgae are of significant importance in the fields of human health, cosmetics, food, and animal feed. In comparison to terrestrial crop plants, microalgae can provide higher productivity and photosynthetic performance, and since they can be cultivated on infertile land, they do not compete with existing food production methods [5,6]. Of the several major production steps of microalgae components, harvesting is both energy and time demanding. It is estimated that microalgae biomass harvesting is responsible for 20–30% of the total biomass production cost [7,8]. Moreover, the harvesting step is crucial for the downstream process, since it leads to a slurry of highly concentrated solid matter [9].

Various harvesting techniques have been introduced over the years such as centrifugation, sedimentation, flocculation, filtration, flotation, and their various combinations. These methods provide (variable) sufficient harvesting efficiencies, however, they are relatively energy and time consuming. Furthermore, none of them can be used as an efficient method for all microalgal cell types, and their application depends on the desired end-product of microalgae cells [10,11]. The reduced lipid levels regarding flocculation, as well as clogging and fouling in filtration, are also considerable disadvantages [12,13]. Moreover, the added cost of the chemicals used in chemical-flocculation or the microorganisms in bio-flocculation, along with the potential contamination of the harvested biomass, are further drawbacks [13,14,15,16].

Magnetic separation, which has been extensively used in the food industry, wastewater treatment, and the steel industry, Refs. [3,17,18] has gathered popularity throughout the past decade as a method for harvesting microalgal cells. Magnetic biomass harvesting, which uses micro- or nano-sized magnetic particles and an applied external magnetic field, is a highly efficient process for cell separation; the method is fast and efficient [17,19,20,21,22]. The process is based on the interactions between the magnetic particles and the surface of the microalgae cells. The magnetic particles can attach to the cells via electrostatic forces, hydrogen bonds, acid-base interactions or van der Waals forces, forming magnetic particle–cell bonding [23]. Key factors affecting the magnetic separation efficiency include cell type and their growth stage, magnetic particle dosage, pH, ions in the cultivation medium, temperature, and gradient of the magnetic field [3]. Both naked and surface-functionalized (using polyacrylamides, chitosan, poly diallyldimethylammonium chloride (PDDA), aminoclay, polyethylenimine (PEI), 3-aminopropyl triethoxysilane (APTES)) magnetic nanoparticles have been applied for the harvesting of *C. reinhardtii*, *C. vulgaris*, *N. salina*, *N. maritime*, *S. dimorfus*, and *S. ovalternus* microalgae, among others [2,3,24,25,26,27,28,29]. Nonetheless, successful upscaling of the magnetic harvesting method depends on some important characteristics of the magnetic particles i.e., their biocompatibility, reusability, and ease of use, as well as the optimization of the parameters affecting the harvesting efficiency [30].

*Chlorella sp.* is a microalga rich in vitamins, polysaccharides, minerals, lipids, and other high-value products [31]. Lutein, a compound with proven anti-cataract properties, is also found in *Chlorella sp.*, while its extracts exhibit anti-tumor, anti-inflammatory, antioxidant, and anti-microbial properties, and reduce blood pressure and cholesterol levels [32,33,34]. As a result, *Chlorella sp.* is one of the most extensively used microalga in cosmetics, pharmaceuticals, food, and animal feed industries [31,35].

Although *Chlorella* *sp.* holds second place in the global “algal products” market [36], its harvesting process is still a major challenge. That is why the harvesting of freshwater microalgae *Chlorella vulgaris* (using microwave-synthesized Fe_3_O_4_ magnetic particles) was examined in this work. The purpose of this study is to develop an efficient *C. vulgaris* harvesting method by examining and optimizing the most important parameters. Specifically, three harvesting parameters have been investigated, namely pH, mass ratio of magnetite particles to biomass, and agitation speed; these were characterized using response surface methodology (RSM). Reusability of the magnetic particles, as well as integrity of the microalgae cells, were also examined. Scanning electron microscopy (SEM) was used to explore the interactions between the microalgae cells and the magnetic particles, while the adsorption mechanism of the *C. vulgaris* cells on the magnetite particles was deduced by measuring the adsorption isotherm at ambient conditions. Finally, zeta potential measurements were performed in order to examine the separation mechanism of *C. vulgaris* microalgae with the aid of the synthesized iron oxide magnetic particles.

## 2. Materials and Methods

### 2.1. Chemical Reagents

For the synthesis of the magnetite (Fe_3_O_4_) particles, iron (II) sulfate (FeSO_4_·7H_2_O) salt (Chem-Lab NV, Zedelgem, Belgium) and sodium hydroxide pellets (NaOH) (Panreac Quimica, SA) were employed. The cultivation medium of the *Chlorella vulgaris* strain was composed of sodium nitrate (NaNO_3_), calcium chloride (CaCl_2_·2H_2_O), magnesium sulfate (MgSO_4_·7H_2_O), potassium hydrogen phosphate (K_2_HPO_4_·3H_2_O), sodium chloride (NaCl), EDTA disodium (Na_2_EDTA), iron (III) chloride (FeCl_3_), manganese (II) chloride (MnCl_2_) and zinc chloride (ZnCl_2_) and cobalt chloride (CoCl_2_) (Sigma–Aldrich, St. Louis, MO, USA). Potassium dihydrogen phosphate (KH_2_PO_4_), vitamin B1, and vitamin B12 provided from Merck (KGaA, Darmstadt, Germany). Deionized water was used for the solubilization of the chemical reagents. Magnetite nano powder (50–100 nm) was purchased from Sigma-Aldrich (St. Louis, MO, USA) for comparison with the microwave-synthesized Fe_3_O_4_ particles used in this work. A trypan blue assay was performed for examining cell membrane integrity (Merck, KGaA, Darmstadt, Germany). All the reagents used were of analytical grade.

### 2.2. Microwave Synthesis of Magnetite (Fe_3_O_4_) Particles

Magnetic particles were prepared using a simple, rapid, and low-cost precipitation method that has been previously reported [20,27]. Briefly, 1 g of FeSO_4_·7H_2_O was diluted in 100 mL of deionized water at ambient temperature. Gradual addition of 1 M NaOH (with continuous magnetic stirring) was used to adjust the pH of the solution to 12. As a result, a black precipitate of Fe(OH)_2_ was formed. By adding deionized water, the final volume of the solution was fixed at 200 mL. The solution was then placed in a common microwave oven where it was radiated for 10 min at 700 W. As a consequence, the Fe(OH)_2_ precipitate was oxidized to Fe_3_O_4_. Subsequently, the magnetite particles were collected by employing a NdFeB magnet (50.8 mm × 50.8 mm × 25.4 mm, Supermagnete, Gottmadingen, Germany) with 12,600–12,900 G (N40) magnetic induction intensity. Thereafter, the Fe_3_O_4_ particles were washed several times with deionized water in order to remove any residual ions, dried in an oven at 60 °C under vacuum, pulverized to a fine powder using a mortar and pestle, and stored in dry conditions for further use.

### 2.3. Microalgal Strain and Cultivation

*Chlorella vulgaris* strain (UTEX 1809) was purchased from Culture Collection of Algae at the University of Texas (Austin, TX, USA). The strain was grown in a medium with the following composition (per liter): NaNO_3_, 250 mg; CaCl_2_·2H_2_O, 25 mg; MgSO4·7H_2_O, 75 mg; K_2_HPO_4_·3H_2_O; 75 mg; KH_2_PO_4_, 175 mg; NaCl, 25 mg; trace element solution, 6 mL from stock solution; vitamin B1, 0.12 μg; vitamin B12, 0.10 μg. One liter of trace element solution contained Na_2_EDTA, 750 mg; FeCl_3_, 97.0 mg; MnCl_2_, 41.0 mg; ZnCl_2_, 5.0 mg; CoCl_2_, 2.0 mg; and Na_2_MoO_4_·2H_2_O, 4.0 mg. The pH was adjusted to 6.8–7.0 and the temperature was controlled at 24 °C. Cell cultures of *C. vulgaris* were grown in 1000 mL Erlenmeyer flasks containing a culture volume of 600 mL at 150 rpm. The injected concentration was adapted to an optical density of 0.1 at 600 nm (OD 600nm). Periodic purity assessment was performed by microscopic examination. The photobioreactor was illuminated at 50 μmol photon m^−2^ s^−1^ with 24/7 white LED lamps at the cell culture surface. *C. vulgaris* growth was monitored by measuring the OD at 600 nm with a UV-VIS S-22 BOECO (Shimadzu, Kyoto, Japan) for up to 15 days. All experiments were conducted in duplicate.

### 2.4. Characterization of Fe_3_O_4_ Particles and Microalgae

#### 2.4.1. X-ray Diffraction of Magnetite Particles and Magnetic Properties

In order to determine the crystallographic structure and phase composition of the synthesized magnetic particles, X-ray diffraction was employed by using a Brooker X-ray D8 advance diffractometer equipped with a Cu-Kα radiation source. The radiation of the specimen was generated at 40 kV and 40 mA at room temperature, with a scanning rate of 0.1° per minute, from 2θ 28° up to 89°. The phenomenon mean diameter size of the magnetite crystallites was calculated via the Debye–Scherrer formula method [37] by exploiting the obtained XRD raw data using the Diffrac. Suite Eva software. Furthermore, the same XRD study was performed for the high purity Fe3O4 nanoparticles purchased from Sigma-Aldrich, in order to compare the two spectra and deduce on the purity and microstructural characteristics of the microwave-synthesized magnetic particles. The magnetic properties of Fe_3_O_4_ were assessed at room temperature using a vibrating sample magnetometer.

#### 2.4.2. Observation with Scanning Electron Microscopy Coupled with Energy Dispersive X-ray Spectroscopy (SEM-EDS)

The morphological characteristics of treated and untreated *C. vulgaris* algal cells, were studied via scanning electron microscopy (SEM) using an FEI Quanta 200 scanning electron microscope.

Using the same technique, the *C. vulgaris*—Fe_3_O_4_ interaction was also evaluated. For this purpose, magnetically harvested *C. vulgaris* cells were fixed in 2.5% (*v*/*v*) paraformaldehyde at 4 °C for 1 h, washed in 0.05 M phosphate buffer (pH 7.4), and consecutively dehydrated in 20%, 35%, 50%, 75%, 90%, and 100% (*v*/*v*) ethanol solution [22].

All the aforementioned samples were sputter-coated with Au prior to SEM examination. Moreover, EDS technique was employed (using an EDAX analyzer) in order to obtain the elemental compositional analysis of the microalgal cells and the magnetite particles.

#### 2.4.3. Microalgae Analysis

Algae concentration (g L^−1^) was calculated using a calibration curve of known optical density at 600 nm and respective dry weights that were determined gravimetrically after drying the algae cells at 60 °C. The optical density was measured with a UV-VIS S-22 BOECO spectrophotometer.

#### 2.4.4. Zeta Potential Measurements

The zeta potential values of the prepared Fe_3_O_4_ particles and the *C. vulgaris* cells were measured with the Zetasizer Nano-ZS (Malvern, UK) at ambient temperature and calculated according to the Smoluchowski equation using the Zetasizer software. In order to perform these measurements, Milli-Q water was used as the dispersion medium for the magnetite particles (3 mg L^−1^) and the microalgae cells (50 mg L^−1^). The pH of the mixtures was fixed to 3.0, 5.0, and 7.0 by adding small quantities of aqueous NaOH and HCl. In each case, at least three zeta potential measurements were recorded, and the reported values correspond to their arithmetic mean.

### 2.5. C. vulgaris Magnetic Harvesting Using Fe_3_O_4_ Particles

#### 2.5.1. Magnetic Harvesting Experimental Procedure

The magnetic harvesting experiments of *C. vulgaris* biomass were carried out under ambient conditions in batch mode. The experimental procedure included the following steps: (a) The pH of the microalgal cultivation was fixed at the desired value through the addition of aqueous NaOH or HCl solutions, (b) in order to facilitate the flocculation between the algal cells and the particles, 10 mL of the microalgae cultivation was mixed with a given amount of magnetic particles for 10 min using a mechanical stirrer operating at desired rpm, and (c) the flocs were separated from the cultivation medium with the application of a strong magnetic field, imposed using the same permanent magnet which was employed during the synthesis of the magnetite particles, for 3 min.

Finally, the harvesting efficiency (*HE*%) of the process was calculated from Equation (1):

(1) $$HE\%=\frac{{\mathrm{OD}}_{0}-{\mathrm{OD}}_{1}}{{\mathrm{OD}}_{0}}\;\cdot 100$$

where, OD_0_ is the initial absorbance of the microalgae cultivation at a wavelength of 600 nm, and OD_1_ is the absorbance at the same wavelength of the supernatant liquid that separates from the microalgae-particles flocs after the application of the magnetic field. The OD_0_ and OD_1_ values were measured with the UV-VIS S-22 BOECO spectrophotometer.

#### 2.5.2. Effect of Harvesting Process Parameters on Microalgae Magnetic Harvesting Using Fe_3_O_4_ Particles

In order to maximize the harvesting efficiency, the effect of the pH of the mixture, the mass ratio (m_r_) of magnetite particles (g)/dry biomass (g) in the algal broth, and the agitation speed (rpm) of the *C. vulgaris* cultivation-Fe_3_O_4_ particles mixture, were examined. Three values of pH (3.0, 5.0, and 7.0), three m_r_ ratios (10:1, 12:1, and 14:1) and three agitation speeds (250, 350, and 450 rpm) were compared. Each separate magnetic harvesting experiment was performed three times, and the reported *HE*% results are the mean values.

Preliminary harvesting experiments aiming to evaluate the effect of the duration of flocs’ magnetic separation were also performed. According to the results, the strong attachment of Fe_3_O_4_ on the cell membrane, along with the application of a powerful magnetic field, results in the rapid harvesting of the flocculated biomass, therefore the study of this parameter was excluded.

#### 2.5.3. Experimental Design for Optimization of *C. vulgaris* Harvesting Using the Response Surface Methodology

The optimum conditions for magnetic harvesting with Fe_3_O_4_ particles were established using central composite design (CCD) under the application of response surface methodology (RSM). The RSM is a combination of mathematical and statistical techniques applicable when several interactive parameters are examined and is considered useful in order to evaluate the relative significance of the operating factors on magnetic harvesting. In the present work, based on preliminary experiments, the independent parameters affecting magnetic harvesting were found to be pH, mass ratio, and agitation speed. Thus, three-level full factorial design (3^k^) was used, considering these operational parameters. The experimental design was devised based on the central level (0) between the minimum (−1) and the maximum levels (+1) of the normalized values. Therefore, twenty experiments were carried out using different values of the examined factors (Table 1): pH (3.0–7.0), mass ratio (10:1–14:1), and agitation speed (250–450 rpm). For a complex system, such as the one under consideration, the response cannot be mathematically expressed through a first-order or a second-order polynomial equation. Therefore, the results of the harvesting experiments were fitted to a modified model, and the quality of the fitted model was quantitatively assessed by the ANOVA method to characterize the interaction effect between independent factors and the microalgae-harvesting efficiency.

Regression analysis and estimation of the coefficients were performed using Design Expert^®^ trial version 12 software.

#### 2.5.4. Adsorption Isotherms

The elucidation of the adsorption capacity and mechanism of the microwave-synthesized magnetite particles on the *C. vulgaris* cells is provided by the adsorption isotherms. In the present study, two different models—Langmuir and Freundlich—were employed in order to analyze the experimental data. These models are frequently reported in related literature as sufficiently accurate to describe the adsorption of magnetic particles on microalgae cells [19,21].

According to the Langmuir model, the amount of dry algae cells adsorbed per unit weight of magnetic particles (*Q_e_* [g g^−1^]), is given by Equation (2):

(2) $$Q_{e}=Q_{m}\cdot \frac{C_{e}\cdot K_{l}}{1+C_{e}\cdot K_{l}}$$

which can be transformed into the following linear form:

(3) $$\frac{C_{e}}{Q_{e}}=\frac{1}{Q_{m}\;\cdot K_{l}}+\frac{C_{e}}{Q_{m}}$$

According to the Freundlich model, *Q_e_* is given by Equation (4):

(4) $$Q_{e}=K_{f}\cdot C_{e}^{\frac{1}{n_{f}}}$$

which can be transformed into the following linear form:

(5) $$lnQ_{e}=lnK_{f}+\frac{1}{n_{f}}\cdot lnC_{e}$$

In Equations (2)–(5), *Q_m_* [g g^−1^] is the maximum adsorption capacity, *C_e_* [g L^−1^] is the concentration of the microalgae cells in the supernatant after completion of the harvesting process, *K_l_* [L g^−1^] is the Langmuir adsorption constant, *K_f_* [g g^−1^] is the Freundlich constant (which is related to the adsorption capacity), and *n_f_* [dimensionless] is the Freundlich factor of the heterogeneity of the adsorption sites.

The adsorption experiments were conducted at ambient temperature in a batch mode in duplicates. For each experiment, an amount of Fe_3_O_4_ was added to 10 mL of algae cultivation of known initial concentration (*C*_0_ [g L^−1^]) in order to realize the magnetic harvesting procedure that is described in Section 2.5.1.

Consequently, the amount of dry algae adsorbed per unit weight of magnetite particles (*Q_e_* [g g^−1^]) was calculated according to Equation (6).

(6) $$Q_{e}=\frac{C_{0}-C_{e}}{m}\cdot V$$

where, *m* [g] is the mass of the magnetic particles used and *V* [L] is the volume of microalgae culture used, i.e., 0.01 L.

#### 2.5.5. Reusability of Magnetic Particles

The study on the reusability of the magnetic particles was realized by carrying out tests with the same (used) particles for cycles, in accordance with the procedure outlined by Markeb et al. [38]. The magnetic particles and microalgae cells obtained from magnetic harvesting were mixed with 10 mL of NaOH (0.5 M) and agitated at 200 rpm for 10 min. The suspension was then sonicated for 10 min. Afterwards, 3 mL of methanol and 3 mL of chloroform were added, and the solution was sonicated for another 10 min. Following sonication, the magnetite particles were collected with a permanent magnet, washed three times, and dried overnight. The detached magnetic particles were used to evaluate the harvesting efficiency tests under the same conditions in order to investigate their reusability.

### 2.6. Statistical Analysis

Tukey’s method, based on one factor ANOVA at the 5% confidence level, was used for the statistical analysis, which was performed with SPSS 15.0.1 software (SPSS Inc., Chicago, IL, USA). Statistically significant differences were reported when the probability of the results (*p*) value is less than 0.05 assuming the null hypothesis.

### 2.7. Cell Membrane Integrity of C. vulgaris Cells

The cell integrity of *C. vulgaris* was determined by the trypan blue staining method. Total of 10 uL of cells were harvested and after addition of 10 uL 1% trypan blue solution the cells were incubated for 10 min at room temperature. The intact cells (viable) remained green (no penetration of the trypan blue solution) while the broken cells appeared blue (stain diffused in the protoplasm).

## 3. Results and Discussion

### 3.1. Characterization of Fe_3_O_4_ Particles

**Figure 1:**
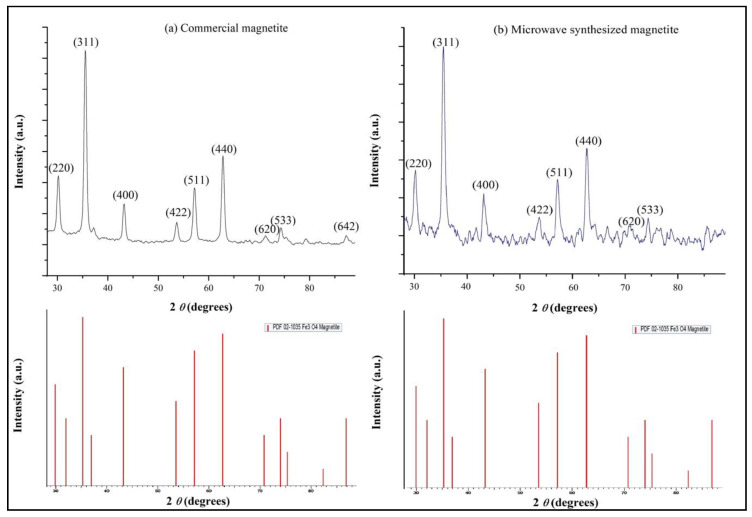
X-ray diffraction spectra of (**a**) commercially available magnetite particles and (**b**) the microwave-synthesized magnetite particles.

Figure 1 demonstrates the X-ray diffraction spectra of the magnetite particles purchased from Sigma-Aldrich (Figure 1a) and the magnetic particles synthesized in this work (Figure 1b). According to the analysis conducted via XRD software Diffrac. Suite Eva, one predominant phase was identified, that of magnetite (PDF 02-1035 Fe_3_O_4_ Magnetite), for both materials.

The noteworthy similarity between the two spectra suggests that they have close microstructural characteristics. Particularly, regarding the microwave-synthesized magnetite (Figure 1b), eight different relatively strong diffraction peaks are displayed in the XRD spectrum at 2 *θ* 30.10°, 35.48°, 42.96°, 53.71°, 57,17°, 62.77°, 70.80°, and 74.27° which correspond to the crystallographic planes (220) (311), (400), (422), (511), (440), (620), and (533) respectively, indicating a cubic inverse spinel structure. Likewise, the same crystallographic planes, can be observed for the commercial magnetite powder (Figure 1a) at 2 *θ* 30.18°, 35.55°, 43.20°, 53.64°, 57,20°, 62.82°, 71.20°, and 74.34°. One more crystallographic plane is present in the spectrum of commercial magnetite at 2 *θ* 87.36°, namely the (642). This last plane cannot be identified with certainty in the XRD pattern of the synthesized magnetite, due to the relatively higher noise encountered. In both cases, it is clearly observed that five diffraction peaks, namely (220), (311), (400), (511), (440) are the main ones. The aforementioned analysis of the Bragg reflections for both magnetite particles was realized by identifying each diffraction peak from the raw XRD data using the Diffrac. Suite Eva software.

Using the tools provided by the same software, the phenomenon mean diameter size of magnetite crystallites was calculated according to the Debye–Scherrer equation [37] for each diffraction peak that was identified. So, an average crystallite diameter size equal to 20 ± 4 nm and 20 ± 3 nm was calculated for the synthesized magnetite and the commercial one respectively.

**Figure 2:**
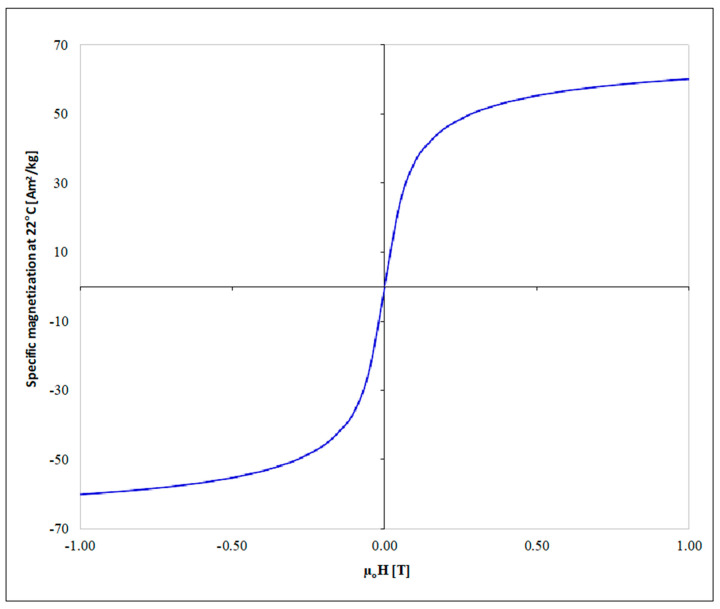
Magnetization curve for the synthesized Fe_3_O_4_.

For the determination of the magnetic properties of the microwave-synthesized magnetite particles, the saturated mass magnetization was determined by vibrating the sample in a magnetometer. The magnetization loop is illustrated in Figure 2. From these results, it is evident that the particles follow a unhysteretic loop, illustrating superparamagnetic behavior at room temperature, thus indicating that their size is below 35 nm [39], which is verified in our case as the particles demonstrate an average diameter of 20 nm. The synthesized magnetic particles (Fe_3_O_4_) exhibit saturation magnetization of 60 emu g^−1^.

Thus, it is concluded that the simple, rapid, and energy-efficient synthesis route followed in this work leads to the formation of high purity nanocrystalline magnetite, which is similar in microstructure to the commercially available that was employed as a means of comparison.

### 3.2. Algae Harvesting Efficiency Optimization

#### 3.2.1. Characterization of *C. vulgaris* and Magnetic Particles Interaction Using SEM

**Figure 3:**
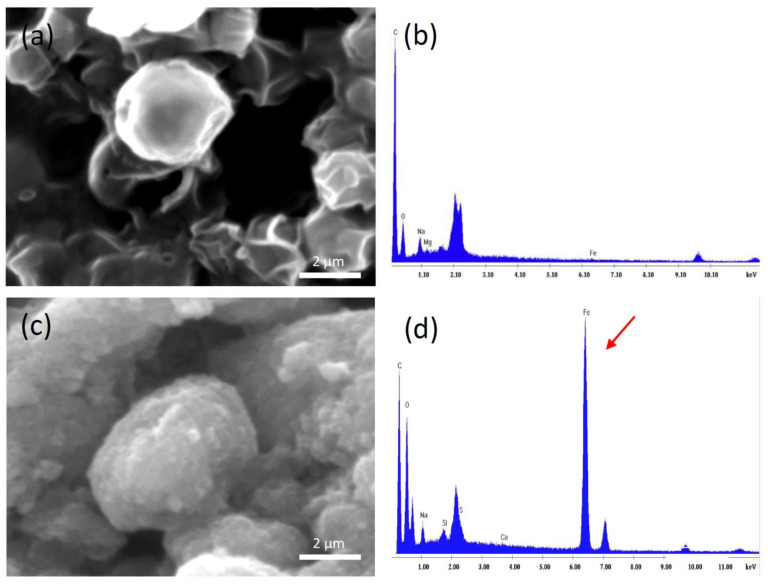
Scanning electron microscopy (SEM) images performed on (**a**) *Chlorella vulgaris* cells before magnetic harvesting, (**b**) EDX analysis for the elemental composition of the cells where the absence of iron peak is reported, (**c**) harvested *Chlorella vulgaris* cells at pH 3.0, (**d**) EDX analysis for the elemental composition of the harvested *Chlorella vulgaris* cells where the iron peak (arrow) is shown.

Evaluation of the interactions between the microalgae and the (synthesized) magnetic particles was tested by characterizing *C. vulgaris* before (control) and after mixing with the magnetic particles using the scanning electron microscopy (SEM). The morphology of *C. vulgaris* cells alone and with magnetic particles, are illustrated in Figure 3. As it can be seen in Figure 3a, the surface of the untreated cells is smooth and spherical, whereas after the magnetic harvesting at pH 3.0 (Figure 3c), it can be observed that all the cell surfaces are fully covered with magnetic particles. The cell surfaces appear rougher and the patterns that are visible in Figure 3a are not discerned in Figure 3c. This confirms that the amount of magnetic particles we chose to use is enough to fully cover every single cell. Moreover, energy dispersive X-ray spectroscopy (EDX) reports the iron peak in Figure 3d, verifying the presence of magnetite on the cells in contrast to the untreated cells (Figure 3b).

#### 3.2.2. Magnetic Harvesting of *C. vulgaris*

A face-centered (distance of each axial point from the center: alpha = 1) composite design was developed for modelling and optimizing (statistically) the magnetic harvesting of *C. vulgaris*. Twenty experiments were carried out examining the effect of pH, mass ratio (m_r_), and agitation speed on harvesting efficiency. The range of the examined parameters was chosen based on preliminary experiments and corresponding literature. Specifically, studies on *Chlorella vulgaris* and related species [2,12,40,41] have been conducted in a pH range between 2 and 12, while maximum harvesting efficiency was achieved in most cases at acidic or neutral pH. The *Chlorella vulgaris* cell walls are very robust and remain resistant at acidic pH, in comparison to other microalgal species that do not attain such a resilient cell wall and might be affected. Although harsh conditions can affect the viability of the cells, the harvesting process followed in this study is very quick, without significantly affecting the biomass composition. Regarding stirring, low agitation speed values [19,42] of around 250 rpm [43], or even higher values at 800 rpm [44] have been used for harvesting *Chlorella* species. This data led us to choose a pH range from 3 to 7 and an agitation speed range between 250 and 450 rpm.

The experimental results are presented in Table 2. It is observed that the decrease of pH, and the increase of the other two parameters, improve the harvesting efficiency. Thus, the best experimental harvesting efficiencies higher than 99% were obtained for pH 3.0 and mixing speed greater or equal to 350 rpm. However, even at a pH similar to that of the cell culture (pH 7.0), the harvesting efficiency is proved to be satisfactory (>85%) for m_r_ = 14:1 and an agitation speed of 450 rpm. These results are in agreement with those reported by Zhu et al. [9] and Bharte and Desai [12], for different *Chlorella* species that demonstrated maximum harvesting efficiency in acidic pH, thus verifying our findings. Analogous studies on *C. vulgaris* harvesting have shown that harvesting based on a flocculation method can lead to high harvesting efficiency, but under different optimum conditions compared to this study. For example, Tork et al. [42] reported a 90% efficiency at basic pH (pH = 11.7) values using cationic starch nanoparticles. Similarly, in the study of Leite et al. [40] for *Chlorella Sorokiniana*, a harvesting efficiency greater than 97% (combined with the appropriate velocity gradient and agitation time) was achieved under highly alkaline conditions (pH = 12). Moreover, a lower optimal agitation rate of 150 rpm was reported by Almomani [19] and Razack et al. [45] (using as a flocculant iron oxide nanoparticles and seed powder of clearing nut respectively). However, the above differences concerning optimal harvesting conditions may be due to the different biomass and magnetic materials, absence or presence of surface modification etc.

Analysis of variance (ANOVA) was used to determine the relationship between the dependent (harvesting efficiency %) and independent variables (pH, mass ratio, and agitation). Transformation of the response was considered necessary in order to bound harvest efficiency within reasonable limits. The transformation chosen is presented below:

(7) $$y^{\prime }=\ln\left(\frac{y-lower}{upper-y}\right)$$

where, *y* stands for the harvesting efficiency (%), lower and upper for the boundaries (40 and 100 respectively), and *y*′ for the transformed harvesting efficiency.

Table 3 shows the statistical results of harvesting efficiency for RSM using ANOVA, while a linear model with respect to the examined factors and their interactions is obtained as follows:

(8) $$y^{\prime }=-2.473-2.022*A+0.299*B+0.027*C-0.003*A*C+0.229*A^{2}$$

where, *y*′ stands for the transformed harvesting efficiency, *A* for pH, *B* for the mass ratio (g_magn. par._/g_biomass_), and *C* for the agitation speed (rpm). It is clarified that through Equation (8) the relation between the independent variables and the transformed dependent variable (*y*′) is expressed. The calculated harvest efficiency (*y*) is finally determined by solving Equation (7) with respect to *y*:

(9) $$y=\frac{100*e^{y^{\prime }}+40}{1+e^{y’}}$$

The model was evaluated based on the F- and *p*-values. For an F-value > 1 and *p*-value < 0.01, the model under consideration is deemed valid. In the present work, both the high F-value (47.58) and low *p*-value (<0.0001) of the model prove its significance and accuracy. All the factors considered were significant (high F-value and *p*-value < 0.05). Specifically, the most important variant is shown to be pH (higher F-value). The obtained value of R^2^ (0.94) implies the high correlation between the dependent and independent variables, while the adjusted R^2^ is only slightly lower (0.93) and in reasonable agreement with the predicted R^2^ (0.86). Finally, the adequate precision (26.60) is higher than 4 indicating that the model can be used to navigate the design space.

**Figure 4:**
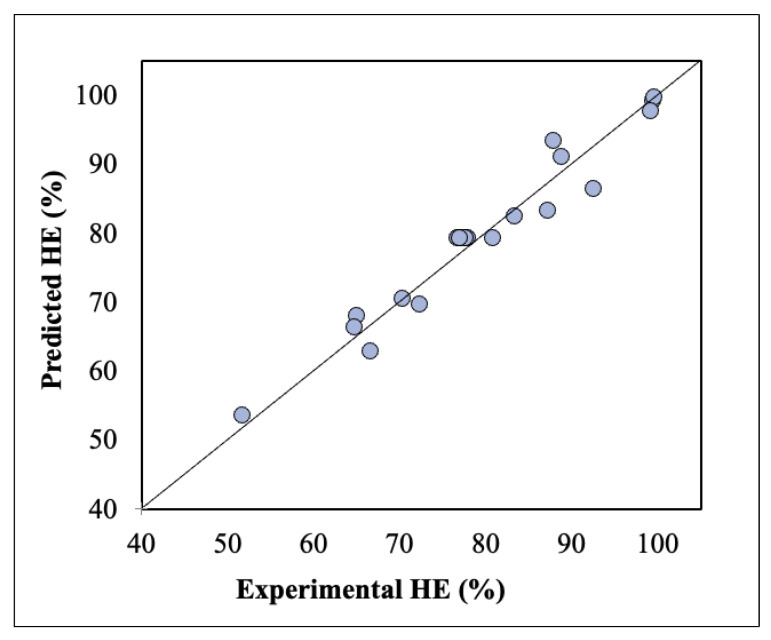
Predicted versus experimental values of harvesting efficiency.

The juxtaposition of the predicted against the experimental values of harvesting efficiency (shown in Figure 4) proves that most of the data points are close to the 45-degree line, proving the reliability of the model.

### 3.3. Separation Mechanism

In order to study the possible electrostatic interactions between the algal biomass and the microwave-synthesized magnetic particles, their zeta potential (ζ) values were measured within the pH range of the magnetic harvesting experiments (pH = 3 to 7). It was found that both the magnetic particles and the biomass cells attain a negative charge. In particular, measurements show ζ values in the range of −17.4 to −33.2, and of −16.6 to −24.9, for the Fe_3_O_4_ particles and the *C. vulgaris* cells respectively, in the specific dispersing medium (Milli-Q water).

**Figure 5:**
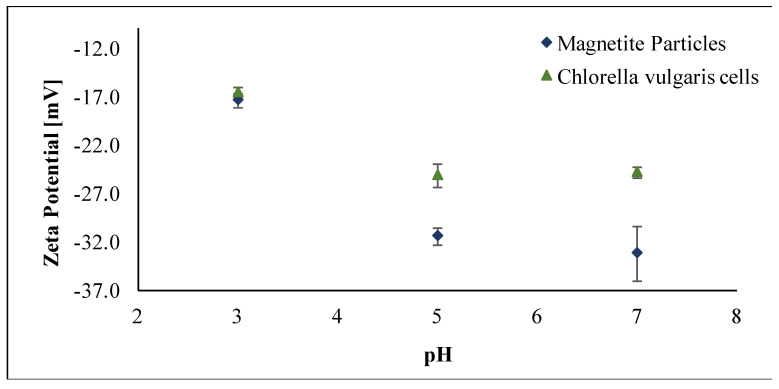
Zeta Potential values of microwave-synthesized Fe_3_O_4_ particles and *C. vulgaris* cells at different pH values of the dispersing medium.

As shown in Figure 5, the zeta potential of the magnetite particles increases with decreasing pH, whereas the *C. vulgaris* cell ζ values remain practically the same as the pH decreases from 7 to 5, and then increases with a further decrease of the pH. Negative surface charge of microalgae biomass is expected in acidic solutions due to the presence of negatively charged groups on the surface of the cells. Concerning the bare Fe_3_O_4_ particles, a net negative surface charge has been previously reported [20,46].

Since in the present study the magnetic particles and the *C. vulgaris* cells are both negatively charged, it could be assumed that the interactions between them are not dependent on a charge neutralization mechanism. Nevertheless, in any aqueous solution, metal oxide (MeOx) particle surfaces can undergo the following protonation/deprotonation reaction:

(10) $$\mathrm{MeOx}—O^{-}\;+\;H_{2}O\;\overset{H_{2}O}{↔}\;\mathrm{MeOx}—\mathrm{OH}\;\overset{H_{2}O}{↔}\;\mathrm{MeOx}—{{\mathrm{OH}}_{2}}^{+}$$

Consequently, under acidic conditions and as the pH decreases, an increase of the positively charged sites on the surface of the metal oxide particles is expected, resulting in an increase in the zeta potential values [28] without necessarily altering the net charge of the particles. This is in agreement with the ζ values obtained here for the microwave-synthesized Fe_3_O_4_ particles. The maximum harvesting efficiency values measured appear at the lowest applied pH values, i.e., at pH = 3. Hence, this behavior may be attributed to local electrostatic interactions between positively charged sites on the surface of the magnetite particles and the negatively charged surfaces of the *C. vulgaris* cells. This assumption is further confirmed by the fact that the harvesting efficiency increases with decreasing pH.

Nevertheless, electrostatic interactions may not be the primary binding mechanism of the synthesized magnetite nanoparticles with *C. vulgaris* cells. Particularly, Fe_3_O_4_ particles can be attached to the microalgal cells through hydrogen bonding; due to the protonation of the magnetite particles under acidic conditions, hydrogen bond donor chemical species OH_2_^+^ formed on Fe_3_O_4_ can interact with hydrogen bond acceptor groups present on *C. vulgaris* cells, such as amino or carboxy groups [47,48]. Furthermore, due to the nano-size of the Fe_3_O_4_ particles, a large specific surface area and high surface energy is expected, characteristics that imply strong adsorption of the particles to the cells [49,50].

### 3.4. Adsorption Isotherms

**Figure 6:**
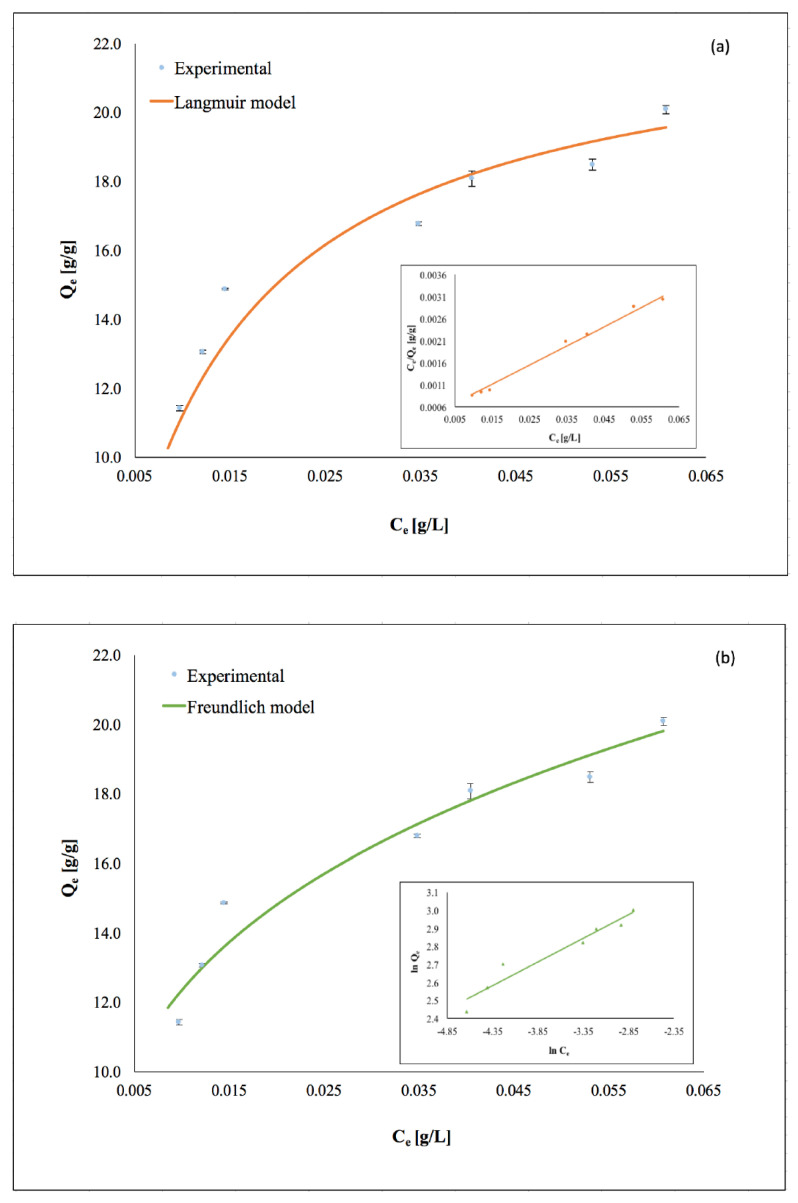
Experimental adsorption isotherm of microwave-synthesized magnetite particles on the *C. vulgaris* cells at 25 °C, fitted with (**a**) Langmuir model and (**b**) Freundlich model; pH = 7, rpm = 450, *C. vulgaris* cell concentration 0.22 g L^−1^.

The adsorption isotherms of the synthesized magnetite particles on the *C. vulgaris* cells are illustrated in Figure 6a,b along with the fitted curves using the Langmuir and Freundlich models respectively. The parameters of both models were obtained using the least squares linear fitting and are presented in Table 4.

The results indicate that the adsorption isotherm of Fe_3_O_4_ particles on the *C. vulgaris* cells is better described by the Langmuir model of adsorption, according to which, a full coverage of the microalgae cells by the magnetic particles occurs. The maximum adsorption capacity, *Q_m_*, is predicted to be equal to 22.95 g of dry biomass per gram of magnetic particles. Moreover, the high *K_l_* value given by the Langmuir model denotes a strong attraction between the *C. vulgaris* cells and the microwave-synthesized magnetic particles [28]. The adsorption mechanism of Fe_3_O_4_ particles on *C. vulgaris* presented here has been previously reported for the same microalgae species both on naked and surface functionalized magnetite particles [19,21].

### 3.5. Regeneration and Reusability of Fe_3_O_4_ Particles

**Figure 7:**
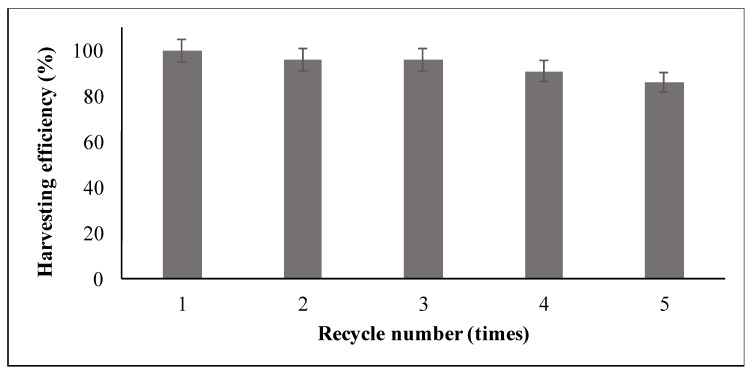
*Chlorella vulgaris* harvesting efficiency (initial concentration of 0.22 g L^−1^) at five different consecutive cycles.

Both microalgae harvesting and the downstream processing of harvested biomass (e.g., to obtain biofuels or bio-products) call for recovery and regeneration of the magnetic particles, alongside testing the cell integrity to ensure the intracellular abidance of bioactive compounds and thereby their efficient and easy extraction. Our data indicate that the magnetic particles can be reused for at least five cycles, with less than 20% decrease of the algae harvesting efficiency—even during the 5th cycle, (Figure 7). This is similar to the results reported by Almomani [19]. These findings indicate that the regeneration procedure followed is an acceptable method, since an efficient reusability of the magnetic particles is observed.

Analogous studies demonstrated a reusability efficiency of 80% after three cycles with chloroform: methanol treatments and ultrasonication [51], and at least 85% after five cycles of acid–base treatment combined with ultrasonication [52]. Markeb et al. [38], using NaOH, methanol, and chloroform as organic solvents, and ultrasonication, reported a minimum 80% recovery of the magnetic nanoparticles. Treatment with NaOH at pH = 12 resulted in 95% recovery for polypropylene/iron oxide nanoparticles, with the recovered magnetic particles retained almost the same microalgae biomass harvesting efficiency as per the newly synthesized ones [53]. An 80% reusability efficiency of cationic surfactant-decorated iron oxide nanoparticles after microalgae detachment using SDS and sonication has also been reported [54].

**Figure 8:**
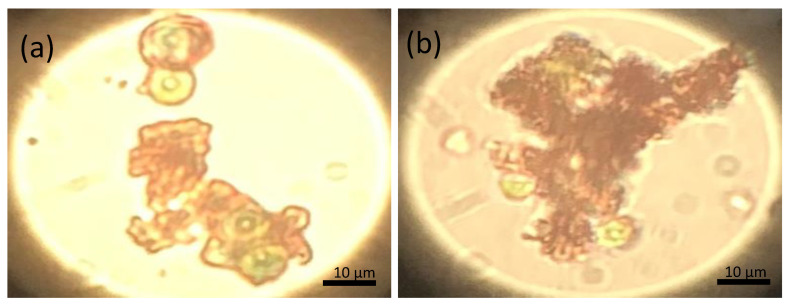
Trypan blue staining (**a**,**b**) on *Chlorella vulgaris* cells bound with magnetic particles (at pH 3.0) indicates the integrity of the cell membrane. Intact cells (viable) remained green, without the penetration of the trypan blue solution, while the broken cells appeared blue as stain diffused in the protoplasm.

Staining with trypan blue (confirming compromised plasma membranes) verified the integrity of the bounded cells on the magnetic particles (Figure 8) and confirmed that no precious intracellular macromolecules were released in the harvesting medium. The trypan blue staining was performed at the most efficient pH for harvesting efficiency (pH = 3). Thus, the cells maintain their ability to produce high value-added products suitable for further use in various biotechnological applications and/or biofuels production.

## 4. Conclusions

*Chlorella vulgaris* harvesting was assessed using iron oxide magnetic particles synthesized using a Fe (II) precursor with the aid of microwave irradiation. The magnetic particles were characterized by X-ray diffraction, identifying one predominant phase, namely that of magnetite, with a mean diameter size of 20 nm. The simple and rapid production route followed in this work leads to the formation of high purity nanocrystalline magnetite with a superparamagnetic behavior at room temperature. The adsorption isotherm of the Fe_3_O_4_ particles on the *C. vulgaris* cells at ambient temperature, demonstrated a full coverage of the microalgae cells by the magnetic particles in accordance with the Langmuir model. Furthermore, the magnetite particles do not impose loss of microalgae cells’ integrity. Response surface methodology verified the experimental optimum operational harvesting conditions, namely pH = 3, mass ratio = 14:1, and agitation speed = 450 rpm, under which a harvesting efficiency equal to 99.6% was achieved. pH was proved as the most crucial variant. The magnetic particles were successfully detached from the microalgal cells and reused for five cycles maintaining at least 80% of their initial harvesting efficiency. The separation mechanism was primarily attributed to the formation of hydrogen bonds between the magnetite particles and the microalgae cells under acidic conditions and to the expression of nano-size effects related to the high surface energy and large specific surface area of the particles. The high magnetic harvesting efficiencies (greater than 99%) obtained at pH 3.0 and mixing speed greater or equal to 350 rpm using microwave-synthesized naked magnetite (Fe_3_O_4_) particles, the cell integrity after the harvesting procedure and the ability to reuse the synthesized magnetic particles for at least five cycles of harvesting, contribute to the novelty of the present work and indicate that the proposed process is efficient and very promising.

## Figures and Tables

**Table 1:** Independent variables and the distribution of their level.

Variables	Level −1	Level 0	Level +1
pH	3	5	7
mass ratio (g_magn._/g_biomass_)	10	12	14
agitation speed (rpm)	250	350	450

**Table 2:** Design table formed by RSM—CCD, presenting the experimental conditions and the experimental values of harvesting efficiency.

Run	pH	Mass Ratio	Agitation Speed (rpm)	Measured Harvesting Efficiency (%)
1	7	10:1	450	64.7
2	3	14:1	450	99.6
3	3	10:1	250	83.3
4	7	14:1	250	72.3
5	7	14:1	450	87.2
6	7	10:1	250	51.7
7	3	10:1	450	99.4
8	3	14:1	250	87.8
9	5	12:1	350	76.9
10	3	12:1	350	99.2
11	5	12:1	450	88.8
12	5	12:1	250	66.5
13	7	12:1	350	65.0
14	5	12:1	350	76.7
15	5	10:1	350	70.3
16	5	12:1	350	77.9
17	5	14:1	350	92.5
18	5	12:1	350	80.8
19	5	12:1	350	77.6
20	5	12:1	350	77.1

**Table 3:** Analysis of variance results for RSM model regarding harvesting process, where *A* is pH, *B* is mass ratio, and *C* is agitation speed.

Source	Sum of Squares	df	Mean	F-Value	*p*-Value
			Square		Prob > F
**Model**	51.99	5	10.40	47.58	<0.0001
**A-pH**	28.47	1	28.47	130.29	<0.0001
**B-mass ratio**	3.58	1	3.58	16.37	0.0012
**C-agitation speed**	12.54	1	12.54	57.39	<0.0001
**AC**	3.21	1	3.21	14.69	0.0018
**A^2^**	4.19	1	4.19	19.15	0.0006
**Residual**	3.06	14	0.22		
**Std. Dev.**	0.47		**R^2^**	0.94	
**Mean**	1.1		**Adj R^2^**	0.93	
**C.V. %**	42.52		**Pred R^2^**	0.86	
**PRESS**	7.96		**Adeq Precision**	26.60	

**Table 4:** Parameters estimated using the Langmuir and Freundlich models.

Langmuir Model			Freundlich Model		
*Q_m_* [g g^−1^]	*K_l_* [L g^−1^]	R^2^	*K_f_* [g g^−1^]	*n_f_*	R^2^
22.95	95.10	0.99	41.30	3.81	0.94

## Data Availability

Additional data for this study are not available on public database, the corresponding author can provide them upon request.
